# Covalency-reinforced oxygen evolution reaction catalyst

**DOI:** 10.1038/ncomms9249

**Published:** 2015-09-10

**Authors:** Shunsuke Yagi, Ikuya Yamada, Hirofumi Tsukasaki, Akihiro Seno, Makoto Murakami, Hiroshi Fujii, Hungru Chen, Naoto Umezawa, Hideki Abe, Norimasa Nishiyama, Shigeo Mori

**Affiliations:** 1 Nanoscience and Nanotechnology Research Centre, Osaka Prefecture University, Osaka 599-8570, Japan; 2 Precursory Research for Embryonic Science and Technology, Japan Science and Technology Agency, Tokyo 102-0075, Japan; 3 Department of Materials Science and Engineering, Osaka Prefecture University, Osaka 599-8531, Japan; 4 National Institute for Materials Science, Tsukuba 305-0044, Japan; 5 Deutsches Elektronen Synchrotron, Hamburg 22607, Germany

## Abstract

The oxygen evolution reaction that occurs during water oxidation is of considerable importance as an essential energy conversion reaction for rechargeable metal–air batteries and direct solar water splitting. Cost-efficient ABO_3_ perovskites have been studied extensively because of their high activity for the oxygen evolution reaction; however, they lack stability, and an effective solution to this problem has not yet been demonstrated. Here we report that the Fe^4+^-based quadruple perovskite CaCu_3_Fe_4_O_12_ has high activity, which is comparable to or exceeding those of state-of-the-art catalysts such as Ba_0.5_Sr_0.5_Co_0.8_Fe_0.2_O_3−*δ*_ and the gold standard RuO_2_. The covalent bonding network incorporating multiple Cu^2+^ and Fe^4+^ transition metal ions significantly enhances the structural stability of CaCu_3_Fe_4_O_12_, which is key to achieving highly active long-life catalysts.

## Abstract (web summary)

Perovskites are cost-effective and efficient catalysts for electrocatalytic oxygen evolution, however often lack stability. Here, the authors report a tetravalent iron based quadruple perovskite which is both active and stable, due to the covalent bonding network incorporating many of its iron and copper ions.

## Graphical abstract

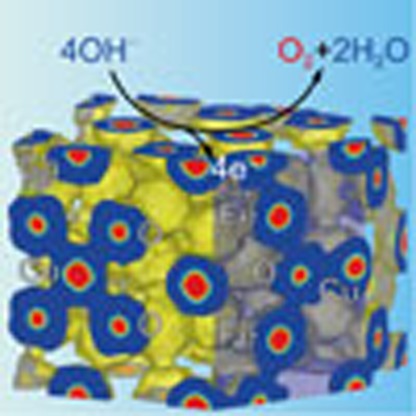

The oxygen evolution reaction (OER: 4OH^−^→O_2_+2H_2_O+4e^−^) is an energy conversion reaction that is essential for both the charging of rechargeable metal–air batteries and direct solar water splitting^1^,^2^,^3^,^4^,^5^. ABO_3_ perovskite oxides are of particular interest because of their high catalytic OER activities, some of which are comparable to those of noble metal oxides such as RuO_2_ and IrO_2_ (refs 6, 7, 8). Along with reports on this high OER activity, many studies have been conducted to clarify the relationship between the electronic state and OER activity in perovskites^6^,^9^,^10^,^11^. Specifically, a simple descriptor of OER activity has been proposed by Suntivich *et al.*^9^; that is, the highest OER activity can be attained when the *e*_*g*_ occupancy of the B-site transition metal is close to unity. Transition metal ions with 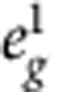 electron configurations enhance the covalency with oxygen ions, leading to effective charge transfer in the rate-determining steps. Cobalt-perovskites such as Ba_0.5_Sr_0.5_Co_0.8_Fe_0.2_O_3−*δ*_ (BSCF) have been widely investigated because of their intrinsically high OER activities, which are consistent with the above descriptor, but surface amorphization in OER cycles remains a serious issue^12^. Therefore, it is necessary to consider the intrinsic catalytic activity and stability separately. In this regard, perovskite oxides containing high-spin Fe^4+^ ions (
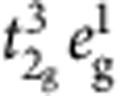
 configuration) such as CaFeO_3_ (CFO) and SrFeO_3_ (SFO) are candidates for OER catalysts with high-catalytic activities. As it is proposed that the electronegativity, which tends to be enhanced in late 3d elements with high valences, serves to increase the metal–oxygen covalency^2^,^13^, it is possible that the Fe^4+^ ions have higher OER activity than the nominally isoelectronic Mn^3+^ ions. The Co^5+^ and Ni^6+^ ions with nominal d^4^ configuration are also expected to have higher OER activity, but the synthesis of perovskite-oxides-containing Co^5+^ and Ni^6+^ ions has not yet been reported. Further, to the best of our knowledge, Fe^4+^-oxides have not been well investigated as OER catalysts to date. This is possibly because of their extreme synthesis conditions, as the majority of Fe^4+^-oxides are synthesized under high pressures of above several GPa. As compounds synthesized under high pressure are metastable, they are likely to be excluded from the promising high-performance catalyst candidates. Thus, no reports on the testing of high-pressure synthesized compounds as electrochemical catalysts have been published^14^. Furthermore, the dissolution of metal ions seems unavoidable, because of the ionic characteristics of A-site alkaline-earth metal ions for AFe^4+^O_3_ perovskites, as in the case of SrRuO_3_, for example, ref. 15.

Recent progress in high-pressure chemistry has enabled dramatic structural modifications, such as transitions from simple A^2+^Fe^4+^O_3_ to quadruple 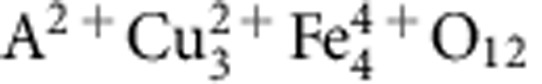 perovskites (A= Ca, Sr; see crystal structures in Fig. 1b). CaCu_3_Fe_4_O_12_ (CCFO) and its analogues exhibit unusual electronic properties, for example, charge disproportionation (2Fe^4+^→Fe^3+^+Fe^5+^)^16^ in the case of CCFO, and the giant negative thermal expansion associated with second-order intersite charge transfer^17^ in the case of SrCu_3_Fe_4_O_12_. The electronic interactions between A′-Cu and B-Fe ions are predominant, where every oxide ion is connected to two B-site ions and one A′-site ion with strong covalency. This is because of the large overlapping that occurs between Cu (Fe) *e*_*g*_ and O 2p orbitals in square-planar (octahedral) coordination. In fact, the electron density distribution of CCFO obtained from our maximum entropy method analysis illustrates a substantial and widespread Fe–O–Cu network (Fig. 1b; details of this electron density analysis are given in the Supplementary Note 1). In contrast, the network is distributed only around Fe and O ions in a simple perovskite SFO. One can expect that the complex covalent bonding network in CCFO plays a significant role in determining its catalytic properties, as in the case of the photocatalytic activity of Pt-loaded CaCu_3_Ti_4_O_12_ (ref. 18). In this report, we show that Fe^4+^-perovskite CCFO exhibits high OER catalytic activity, which is comparable to or exceeds that of state-of-the-art OER catalysts such as BSCF and the gold standard RuO_2_. CCFO also possesses high stability under OER conditions over many cycles, owing to its enhanced covalent bonding network.

## Results

### Catalytic activity of Fe^4+^-perovskites

The OER catalytic performance of the Fe^4+^-perovskites CFO, SFO and CCFO is compared with that of BSCF and RuO_2_, together with a nominally isoelectronic perovskite, LaMnO_3_ (LMO), in Fig. 2. The tetravalency of the Fe ions for CCFO was confirmed via Fe K-edge X-ray absorption spectra (Supplementary Fig. 2). To exclude geometrical effects, the current density per oxide surface area 
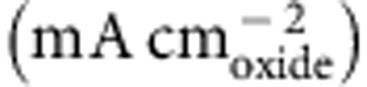
, in which the surface areas were determined using Brunauer–Emmett–Teller (BET) analysis, was adopted as the vertical axis in the voltammograms in this study (Supplementary Note 2, Supplementary Fig. 3, and Supplementary Table 1). As the OER activity of BSCF is strongly dependent on the synthesis conditions^9^,^12^,^19^,^20^, two different BSCF samples calcined at 950 and 1,100 °C (BSCF_950_ and BSCF_1100_, respectively) were tested.

Figure 2a shows the obtained linear sweep voltammograms, and it can be seen that CCFO exhibits the highest OER activity of the catalysts tested here. The overpotential of CCFO for OER (*η*=0.31 V), which was determined based on the onset potentials at 0.5 mA 
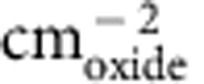
, is the lowest of the examined substances, while its specific activity (current density at 1.6 V versus RHE) is the highest (Fig. 2a,b). The CCFO Tafel slope (51 mV dec^−1^) is as low as those of the SFO and CFO (63 and 47 mV dec^−1^, respectively; Fig. 2c). The excellent properties of CCFO, which are attributed to the presence of the Fe^4+^ ions, exceed those of BSCF_1100_ and RuO_2_. On the other hand, the BSCF performance reported by Suntivich *et al.*^9^ is superior to that of the CCFO examined in this study. This is attributed to the difference in the synthesis conditions of these particular samples, because BSCF has exhibited different OER performance in a number of reports^9^,^12^,^19^,^20^ (see also, the XRD profiles of the tested BSCF samples in Supplementary Fig. 4). Thus, we are unable to definitively conclude that CCFO exhibits superior OER performance to BSCF in this study, and further investigations are required in order to compare the intrinsic OER activities of these substances. However, the intrinsic superiority of the Fe^4+^ ions can be confirmed by comparison between AFe^4+^O_3_ (A=Ca, Sr) and non-Fe^4+^ oxides. The overpotentials of SFO and CFO are 0.41 and 0.39 V, respectively, which are comparable to those of BSCF (*η*=0.38 and 0.36 V for BSCF_950_ and BSCF_1100_, respectively) and are lower than that of RuO_2_ (*η*=0.49 V). By comparing the specific activities (current densities at 1.6 V, Fig. 2b), it can be seen that SFO and CFO have high activities comparable to those of BSCF_1100_ and RuO_2_. On the other hand, LMO exhibits poor OER catalytic activity; its overpotential cannot be defined because of the overly small current density, while the specific activity is only a centesimal fraction of that exhibited by AFeO_3_. This is because the secondary descriptor, electronegativity^2^,^13^, predominates the OER activity in these cases.

As the B-site ion (Fe^4+^) is identical among CFO, SFO and CCFO, the excellent OER activity of CCFO can be attributed to its particular structure, that is, it is a quadruple perovskite incorporating ordered A′-site Cu ions. It should be noted that a reference Cu^2+^–Fe^3+^ complex oxide examined in this study, CuFe_2_O_4_ spinel, did not exhibit high OER activity (Supplementary Fig. 5), thus, the combination of a Cu^2+^O_4_ square and Fe^4+^O_6_ octahedron is a key factor enhancing the OER activity, as will be discussed below.

### Stability of Fe^4+^-perovskites

**Figure 1:**
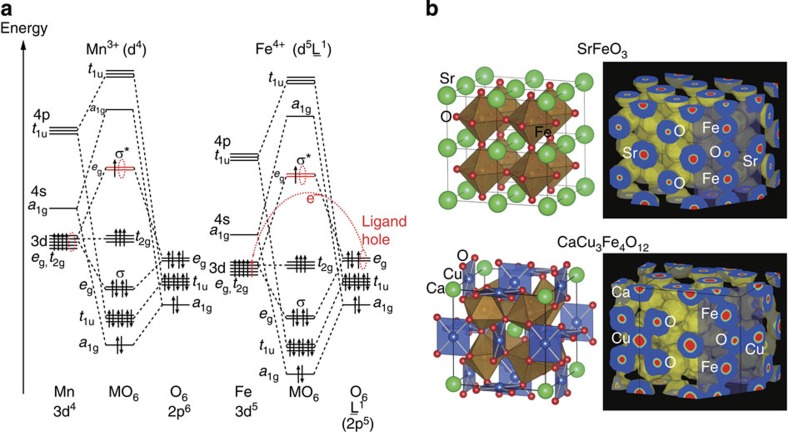
Electronic and crystal structures of SFO and CCFO perovskites. (**a**) Schematic illustration of molecular orbitals for regular Mn^3+^O_6_ and Fe^4+^O_6_ octahedra. The Mn^3+^- and Fe^4+^- ion 3d-orbital energy levels are higher and lower than those of the O 2p orbitals, respectively. Therefore, the highest occupied molecular orbitals σ* generated from the e_*g*_ and 2p orbitals have 3d and 2p characteristics for the Mn^3+^ and Fe^4+^ ions. The holes at the σ* orbitals are due to the e_*g*_ and O 2p orbitals in the former and latter, respectively, resulting in different representations of d^4^ and d^5^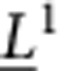 for Mn^3+^ and Fe^4+^, respectively, where 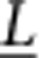 denotes a ligand hole at the O 2p orbital^25^. The *π*-bonds between the t_2*g*_ and 2p orbitals are neglected for simplicity. (**b**) Crystal structures and 3D electron density maps of SFO and CCFO. SFO is crystallized in a cubic ABO_3_-type perovskite structure, and CCFO is crystallized in a cubic quadruple AA′_3_B_4_O_12_-type structure with a 2*a*_0_ × 2*a*_0_ × 2*a*_0_ unit cell (*a*_0_: *a*-axis length of a simple ABO_3_ perovskite). In these types of perovskites, the A-sites are occupied by alkaline, alkaline-earth or rare-earth metal ions, the A′-sites by Jahn–Teller active ions such as Cu^2+^ and Mn^3+^, and the B-sites by d-block transition metal ions. 3D electron density maps of SFO (equi-density level: 0.4 Å^−1^) and CCFO (equi-density level: 0.5 Å^−1^) were obtained from maximum entropy method analysis of synchrotron X-ray powder diffraction data. The shaded cross-sections indicate the (110) and 
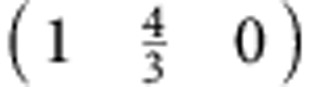
 planes of SFO and CCFO, respectively. The widespread covalent network incorporating the Cu, Fe and O ions is exemplified by CCFO. These illustrations were drawn using the VESTA3 program^26^. The synchrotron X-ray powder diffraction patterns and Rietveld refinement results are shown in Supplementary Fig. 1 and Supplementary Note 1.

**Figure 2:**
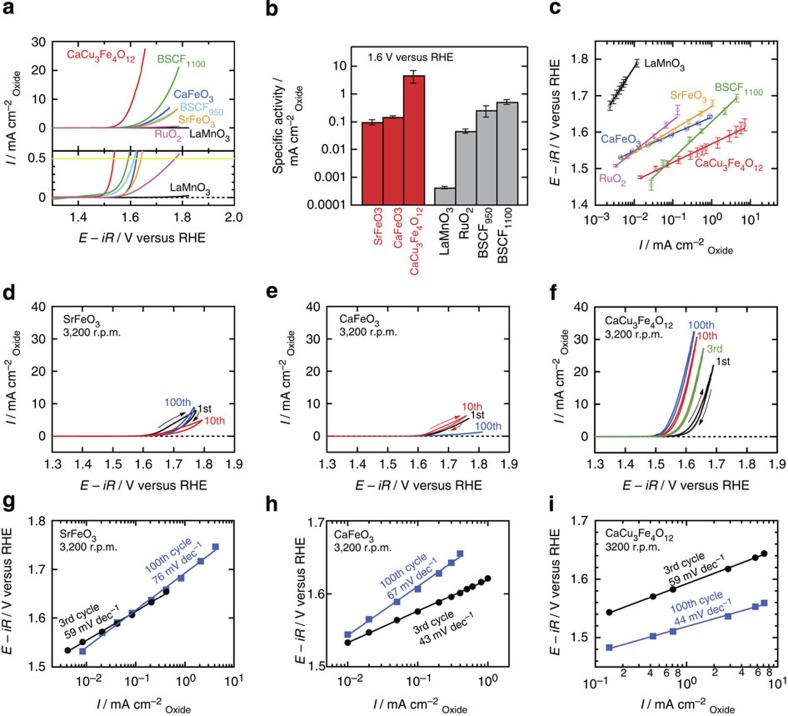
OER catalytic performance of Fe^4+^-perovskites and references. (**a**) Linear sweep voltammograms for OER for SFO, CFO, CCFO, LMO, BSCF and RuO_2_. The overpotential (*η*) of each catalyst was determined from the onset potential, *E*_onset_ (V versus RHE); *E*_*onset*_ is the potential at 0.5 mA 
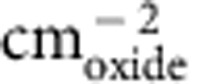
 and *η*=*E*_onset_−1.23 (V). (**b**) Specific activities (current density at 1.6 V versus RHE) for SFO, CFO, CCFO, LMO, BSCF and RuO_2_. (**c**) Tafel plots for SFO, CFO, CCFO, LMO and BSCF. The error bars show the s.d. of three independent measurements. All data in (**a**–**c**) were obtained from the third cycle. Cyclic voltammograms of (**d**) SFO, (**e**) CFO and (**f**) CCFO for 100 cycles. Cycle dependence of Tafel slopes for (**g**) SFO, (**h**) CFO and (**i**) CCFO. Hundred continuous cycle measurements were performed with a higher disk rotation rate of 3,200 r.p.m. to prevent adhesion of the O_2_ bubbles to the electrode. In (**b**,**c**) the error bars correspond to the s.d. obtained from three independent measurements.

The Fe^4+^-perovskite stability under OER conditions was tested. Fig. 2d–f show the cyclic voltammograms (CV) of SFO, CFO and CCFO for continuous 100 cycles. In the SFO case, the OER current density is suppressed even in the anodic sweep of the first cycle, because of the degradation of the SFO; this implies the dissolution of metal ions^15^. On the other hand, the increase in current density for 100th cycle is possibly attributed to the increase in electrochemical surface area in amorphization^12^. For the CFO, the OER current density increases slightly in the first ∼10 cycles, and then gradually decreases over the 100 cycles. As can be seen in the Tafel plots of the 3rd and 100th cycles for the SFO and CFO (Fig. 2g,h), both the Tafel slopes are increased after 100 cycles; this clearly suggests the degradation of the SFO and CFO under OER conditions (also see the increases in overpotentials for 100 cycles in Supplementary Note 3 and Supplementary Fig. 6). Considering the fact that the highest occupied molecular orbitals are dominated by the O 2p orbitals in SFO and CFO, as shown in Fig. 1a, the above results are explained by the trend that the highly elevated O 2p band centre (or the deep Fe 3d orbital) increases the activity but decreases the stability, as suggested for cobalt perovskites^6^. However, CCFO is remarkably stable up to 100 cycles, in spite of the fact that it has the same electronic configuration as SFO and CFO. For CCFO, the current density increases in the first ∼10 cycles and remains almost unchanged. The CCFO Tafel slope does not vary significantly, rather it improves slightly over the 100 cycles (Fig. 2i). This corresponds to a slight improvement in the catalytic activity. Thus, we conclude that CCFO is an excellent OER catalyst that satisfies both the activity and stability requirements.

**Figure 3:**
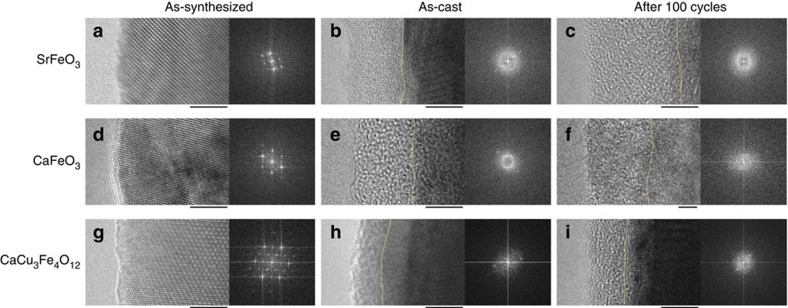
HRTEM and fast fourier transform (FFT) images of perovskite oxides before and after OER measurements. The boundaries between the crystalline and amorphous regions are divided by orange dotted lines. All the FFT images were obtained from surface regions of ∼10 × 10 nm^2^. Scale bar: 5 nm.

To determine the difference in stability for these Fe^4+^-perovskites, the surface structures of SFO, CFO and CCFO were investigated using high-resolution transmission electron microscopy (HRTEM) both before and after the 100-cycle OER measurements. Figure 3 shows surface HRTEM images of SFO, CFO and CCFO samples, as-synthesized, as-cast, and after the 100-cycle OER measurements. Well-crystalline surface structures can be observed for all the as-synthesized powders and the bulk crystallinity of all the samples was retained after the 100-cycle OER measurements (see also, the electron diffraction patterns in Supplementary Fig. 7). However, thin amorphous layers (∼5 nm) formed on the surfaces of all the as-cast catalysts. Further amorphization gradually occurred during the OER cycles in the case of the SFO and CFO samples, resulting in thick amorphous layers about 20 nm after 100 cycles. These amorphous layers caused suppression of the OER reaction of these two perovskites with decreased current density as shown in Fig. 2d,e. In contrast, CCFO retained the thin amorphous layer (∼5 nm) even after 100 cycles, and no erosion was observed. If the amorphous layers isolated only the catalyst surfaces from the electrolyte, the catalytic activities would converge on the lower levels equally. However, the experimental results suggest that the amorphous layers reflect the bulk properties to some extent. The significant improvement in the CCFO stability in comparison with that of AFe^4+^O_3_ can be attributed to the transformation of the covalent bonding networks. In a simple AFe^4+^O_3_ perovskite, the B-site Fe^4+^ ions are covalently bound to the oxide ions, whereas the A-site alkaline-earth metal ions have ionic characteristics^15^. Thus, the A-site ions are easily dissolved in the electrolyte during OER^12^. In contrast, the covalent Cu–O bonds in square-planar units of CCFO aid formation of the covalent bonding network and prevent progressive amorphization during the OER measurements.

## Discussion

**Figure 4:**
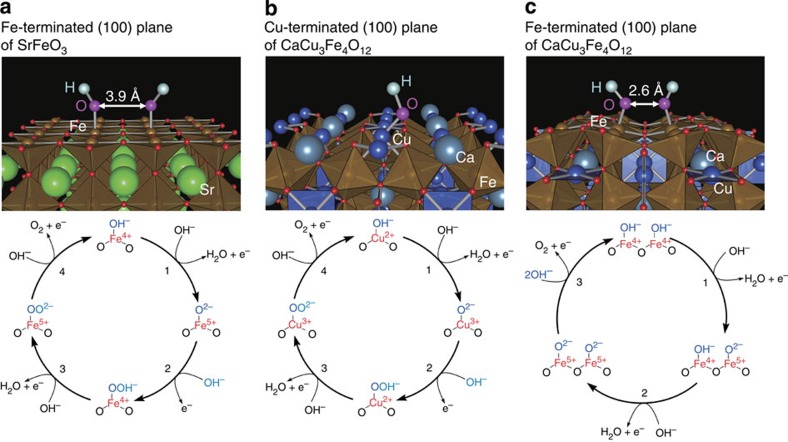
OH^−^ adsorbed surfaces for SFO and CCFO and corresponding OER mechanism. (**a**) OH^−^ adsorbates on Fe-terminated (100) plane of SFO for Fe-mediated route (ER type). The interatomic distance between the nearest neighbouring OH^−^ adsorbates is ∼3.9 Å. (**b**) OH^−^ adsorbate on (Ca,Cu)O-terminated (100) planes of CCFO for Cu-mediated route (ER type). (**c**) OH^−^ adsorbates on FeO_2_-terminated (100) planes of CCFO for Fe-mediated route (LH type). The interatomic distance between the nearest neighbouring OH adsorbates is ∼2.6 A. In all cases, the Cu^2+^/Cu^3+^ or Fe^4+^/Fe^5+^ redox couple acts as the reaction mediator under the assumption that the adsorbed OH^−^ ions occupy the original oxygen sites in the crystal structure.

Here, we demonstrate the structural features of CCFO that are associated with OER catalytic activity. When we assume that the local crystal structures of CCFO are reflected on the surface at a certain level, several factors that increase the catalytic activity are considered. Fig. 4 proposes three possible OER routes for SFO and CCFO. The left route is the conventional Eley-Rideal (ER)-type mechanism for SFO and CCFO. In the ER-type mechanism, OH^−^ adsorbates are bound to B-site Fe ions on the surface (Fig. 4a), in which the rate-determining step is considered to be the formation of the O–O bond (reaction 2) or the subsequent deprotonation (reaction 3)^9^ along with the redox reaction of the B-site ions. In both cases, the electron charges are transferred to the Fe ions efficiently through strong Fe–O covalent bonds. An almost identical mechanism is most likely valid on the Cu-terminated surface of CCFO (Fig. 4b). The Cu and Fe ions can tolerate the redox reactions in the Cu^2+^/Cu^3+^ and Fe^4+^/Fe^5+^ states, leading to the stable and high OER activity exhibited by CCFO. It should be noted that the Langmuir-Hinshelwood (LH)-type reaction can occur through the direct formation of the O–O bond between the neighbouring oxygen atoms connected to the nearest neighbouring Fe ions, because of the short distance (Fig. 4c). The oxygen–oxygen distance is shortened to ∼2.6 Å by heavily bent Fe–O–Fe bonds (∼140°) for CCFO. This oxygen–oxygen distance is comparable to that of *α*-Mn_2_O_3_, in which the LH-type mechanism is thought to dominate^21^. In contrast, the oxygen–oxygen distances for simple cubic perovksite SFO is ∼3.9 Å because of the linear Fe–O–Fe bonds (=180°). This oxygen–oxygen distance is too large to permit the oxygen atoms to interact with each other and form oxygen molecules. In the LH-type reaction, one of the two possible rate-determining steps in the ER-type reaction (that is, the deprotonation of the oxyhydroxide group to form peroxide ions) is skipped, resulting in the acceleration of the reaction. Thus, the realization of the LH-type reaction is another major specificity of CCFO.

In summary, the Fe^4+^-perovskite CCFO exhibits promising OER activity. Further, CCFO has a widespread covalent bonding network that enhances its stability. The cationic arrangements of this substance provide a further increase in the OER activity. These findings indicate that the covalent network consisting of multiple transition metal ions in CCFO plays a crucial role in the activity and stability of the OER catalysis. In addition, the various unexplored A′-B ion couplings in quadruple perovskites may provide further high-performance, high-stability and cost-effective OER catalysts.

## Methods

### Sample preparation

SFO, CFO and CCFO were synthesized via a high-pressure synthesis method. LMO and BSCF were obtained using a polymerized method, while CuFe_2_O_4_ was synthesized via the inverse coprecipitation method. RuO_2_ (99.9%) was used as purchased from RARE METALLIC, Co, Ltd. The sample preparation details are given in the Supplementary Methods.

### Characterization

X-ray diffraction patterns of reference oxides were obtained using a laboratory X-ray diffractometer (Rigaku Ultima IV) with Cu Kα radiation. Synchrotron X-ray powder diffraction patterns of the Fe^4+^-perovskites were obtained at the SPring-8 BL02B2 beamline. Fe K-edge X-ray absorption spectra of the CCFO and Fe^3+^ reference oxides were collected at room temperature and in absorption mode at the SPring-8 BL01B1 beamline. Crystal structure refinements of CFO, SFO and CCFO were conducted based on the obtained synchrotron X-ray powder diffraction data using a Rietveld refinement program RIETAN-FP^22^. Electron density analysis of SFO and CCFO was performed using the Dysnomia maximum entropy method program^23^. HRTEM images were collected using a JEOL JEM-2100F.

### Preparation of catalyst inks

The catalyst inks were prepared by reference to the methods reported by Suntivich *et al.*^9^,^24^ and Jung *et al.*^7^ K^+^ ion-exchanged Nafioni was used as a immobilizing binder, which did not prevent the transport of dissolved O_2_ to the catalyst surface. A ∼3.33 wt.% K^+^ ion-exchanged Nafion suspension was prepared by mixing a 5 wt.% proton-type Nafion suspension (Sigma-Aldrich) and 0.1 M KOH aqueous solution at 2:1 by volume. The pH of the 5 wt.% proton-type Nafion suspension was initially ∼1 and 2 and was changed to ∼11 after mixing. The catalyst inks of the perovskites and reference oxides (RuO_2_ and IrO_2_, Sigma-Aldrich) were prepared by mixing 50 mg of oxide, 10 mg of acetylene black (AB), and 0.3 mL of ∼3.33 wt.% K^+^ ion-exchanged Nafion suspension. The volumes of the inks were adjusted to 10 mL by the addition of tetrahydrofuran (Sigma-Aldrich). Thus, the final concentration of the catalyst inks was 5 mg_oxide_ 
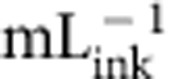
, 1 mg_AB_ 
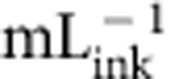
 and ∼1 mg_Nafion_ 
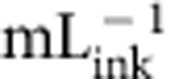
. A rotating ring-disk electrode (BAS Inc, Japan) consisting of a glassy carbon (GC) disk of 0.4 cm in diameter and a Pt ring part of 0.7 and 0.5 cm outer and inner diameter, respectively, was used as a working electrode after mirror polishing with 0.05 μg alumina slurry (BAS Inc). Then, 6.4 μL of catalyst ink was drop-cast onto the GC disk part (0.2 × 0.2 × π cm^2^). The catalyst layer on the GC disk part was dried overnight in vacuum at room temperature, and was composed of 0.25 mg_oxide_ 
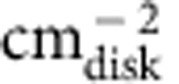
, 0.05 mg_AB_ 
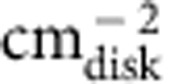
 and ∼0.05 mg_Nafion_ 
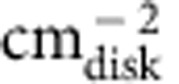
.

### Electrochemical characterization

Electrochemical characterization was conducted with a rotating-disk electrode rotator (RRDE-3 A, BAS Inc) at an electrode rotation rate of 1,600 or 3,200 r.p.m. in combination with a bipotentiostat (ALS Co, Ltd., Japan). For all experiments, a Pt wire electrode and Hg/HgO electrode (International Chemistry Co, Ltd., Japan) filled with a 0.10 M KOH aqueous solution (Nacalai Tesque, Inc, Japan) were used as the counter and reference electrodes, respectively. All measurements were conducted under O_2_ saturation at room temperature (∼25 °C ), which fixed the equilibrium potential of the O_2_/H_2_O redox couple to 0.304 V versus Hg/HgO (or 1.23 V versus RHE). For the catalysis evaluation of the perovskites for OER, the potential of the catalyst-modified GC part was controlled from 0.3–0.9 V versus Hg/HgO (1.226–1.826 V versus RHE) at 10 mV s^−1^. For all measurements, the current density was *iR*-corrected (*R*=∼43 Ω) using the measured solution resistance, and capacitance-corrected by taking the average between the anodic and cathodic scans^9^. All the OER currents are shown relative to the surface area of the oxide catalysts estimated using BET analysis (BELSORP-max, BEL Japan, Inc, Japan).

## Additional information

**How to cite this article:** Yagi, S. *et al.* Covalency-reinforced oxygen evolution reaction catalyst. *Nat. Commun.* 6:8249 doi: 10.1038/ncomms9249 (2015).

## Supplementary Material

Supplementary Information Supplementary Figures 1-7, Supplementary Table 1, Supplementary Notes 1-3, Supplementary Methods and Supplementary References
