# Supplementary material for: Covalency-reinforced oxygen evolution reaction catalyst
Source: Nat Commun. 2015 Sep 10;6:8249. doi: 10.1038/ncomms9249 (PMC4579779; doi:10.1038/ncomms9249)
Supplement: Supplementary Information — Supplementary Figures 1-7, Supplementary Table 1, Supplementary Notes 1-3, Supplementary Methods and Supplementary References [file ncomms9249-s1.pdf]

Supplementary Figures

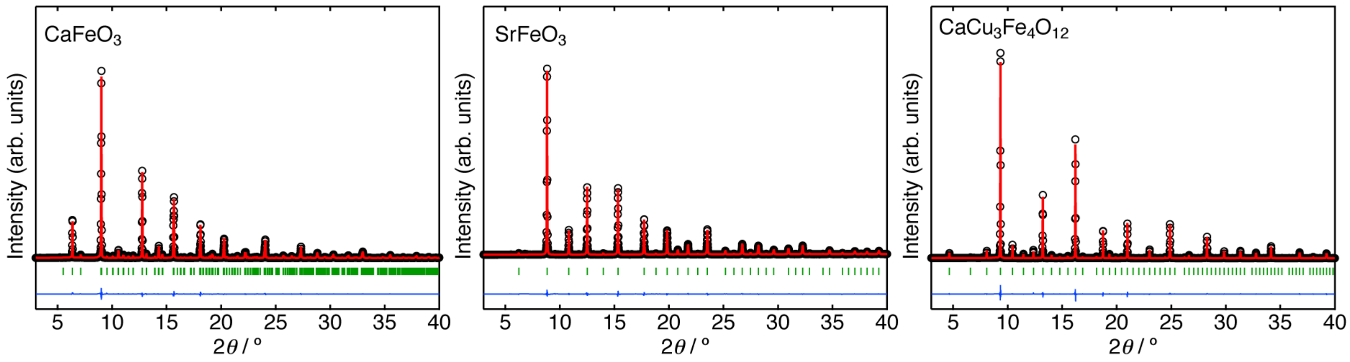

Supplementary Figure 1: **Observed synchrotron XRD profiles and Rietveld refinement results for as-synthesized perovskite oxides.** The green vertical marks indicate the Bragg reflection positions, while the black circles and red lines indicate the observed and calculated profiles, respectively. The bottom blue curve displays the difference between the observed and calculated profiles. $\lambda = 0.41990$ Å.

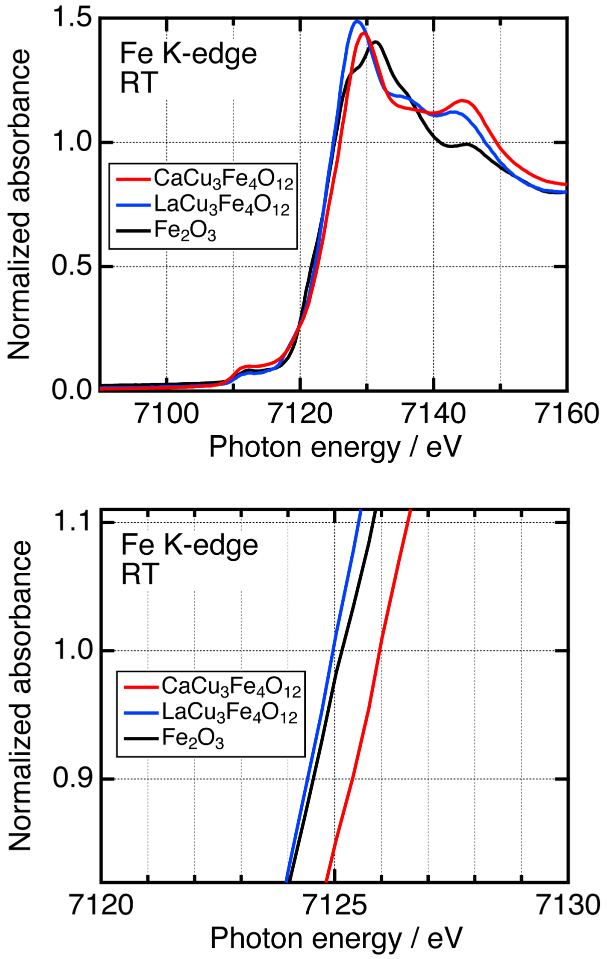

Supplementary Figure 2: **X-ray absorption spectra of $CaCu_3Fe_4O_{12}$.** $CaCu_3Fe_4O_{12}$ exhibits a significant edge shift by ∼1 eV compared to the Fe^{3+} reference oxides. This edge-shift value is comparable to that (∼1.3(2) eV)[1] between $LaFe^{3+}O_3$ and $SrFe^{4+}O_3$, confirming that the valence state of $CaCu_3Fe_4O_{12}$ at room temperature is close to $CaCu^{2+}{}_3Fe^{4+}{}_4O_{12}$.

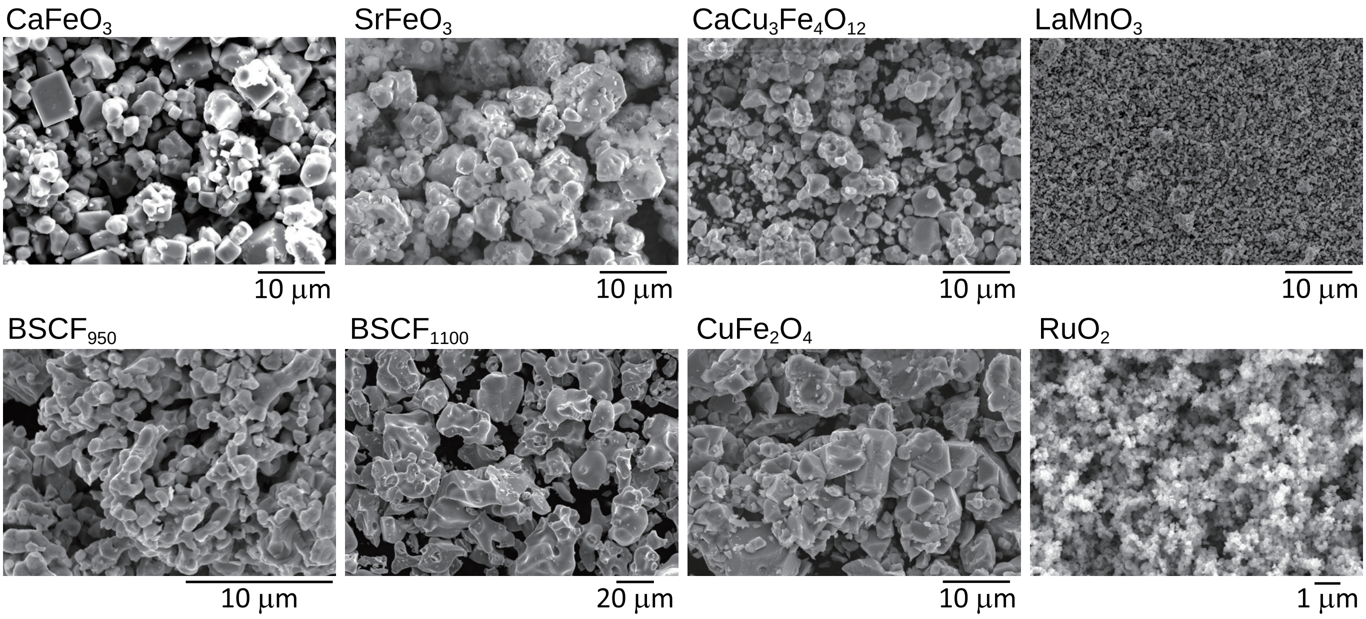

Supplementary Figure 3: **SEM images of catalyst oxides.**

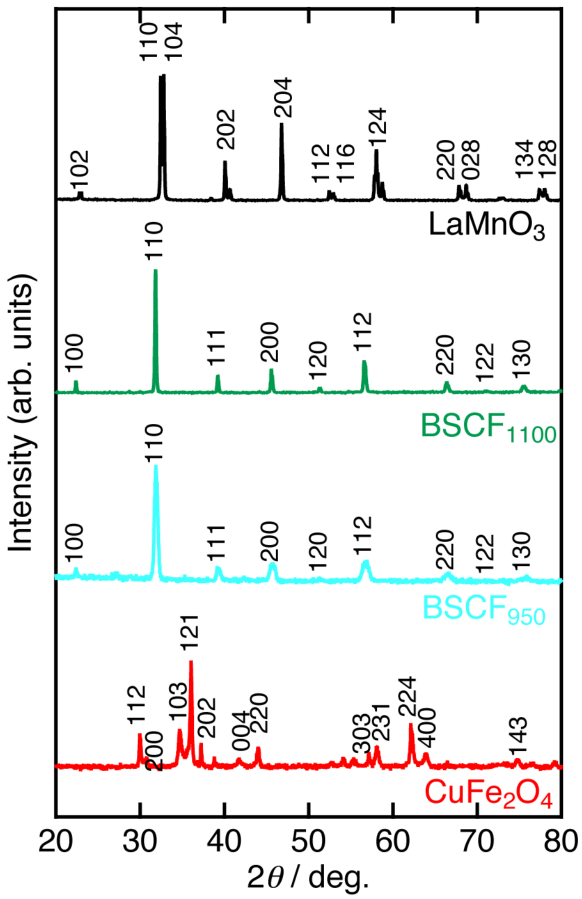

Supplementary Figure 4: **XRD patterns of reference catalysts collected with Cu Kα radiation.** The XRD pattern of $LaMnO_3$ was indexed with a rhombohedral perovskite structure (space group: $R\bar{3}c$) with $a = \sim5.52$ Å and $c = \sim13.31$ Å. The XRD patterns of $BSCF_{950}$ and $BSCF_{1100}$ were indexed with a cubic perovskite structure (space group: $Pm\bar{3}m$) with $a = \sim3.98$ Å, and the XRD pattern of $CuFe_2O_4$ was indexed with a tetragonal spinel structure (space group: $I4_1amd$) with $a = \sim5.82$ Å and $c = \sim8.69$ Å.

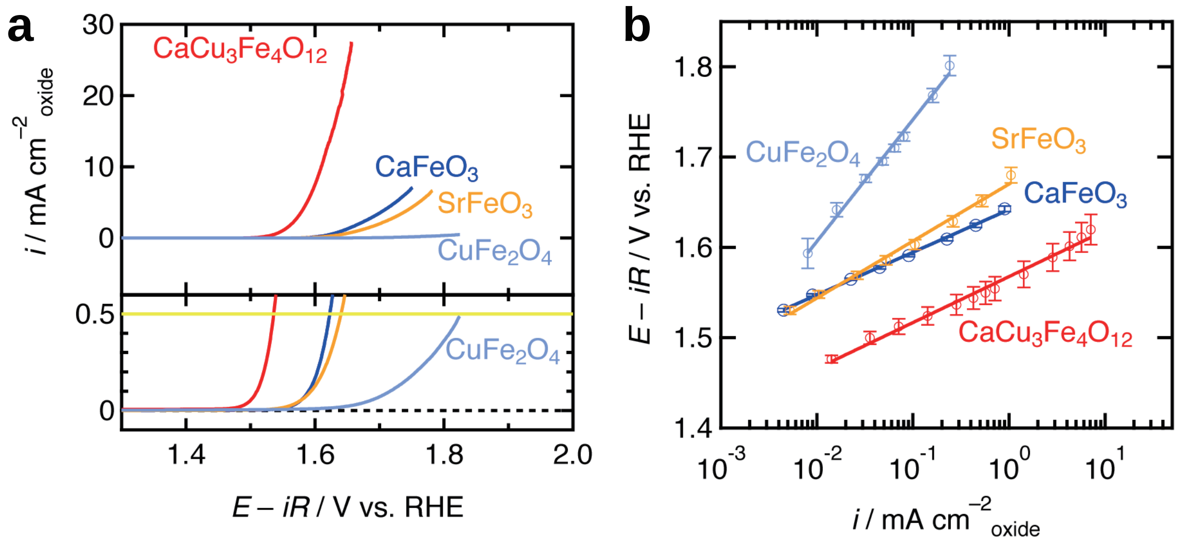

Supplementary Figure 5: **OER activity of $CuFe_2O_4$ spinel oxide.** **(a)** Linear sweep voltammogram of $CuFe_2O_4$ compared with those of $CaFeO_3$, $SrFeO_3$, and $CaCu_3Fe_4O_{12}$. **(b)** Tafel plot of $CuFe_2O_4$ compared with those of $CaFeO_3$, $SrFeO_3$, and $CaCu_3Fe_4O_{12}$. The measurements were conducted at an electrode rotation rate of 1,600 rpm and a potential sweep rate of 10 mV s^{-1} in a 0.10 M KOH aqueous solution under O_2 saturation at room temperature (approximately 25 °C). The Tafel slope of $CuFe_2O_4$ is 135 mV/dec, which is significantly larger than those of $CaFeO_3$, $SrFeO_3$, and $CaCu_3Fe_4O_{12}$.

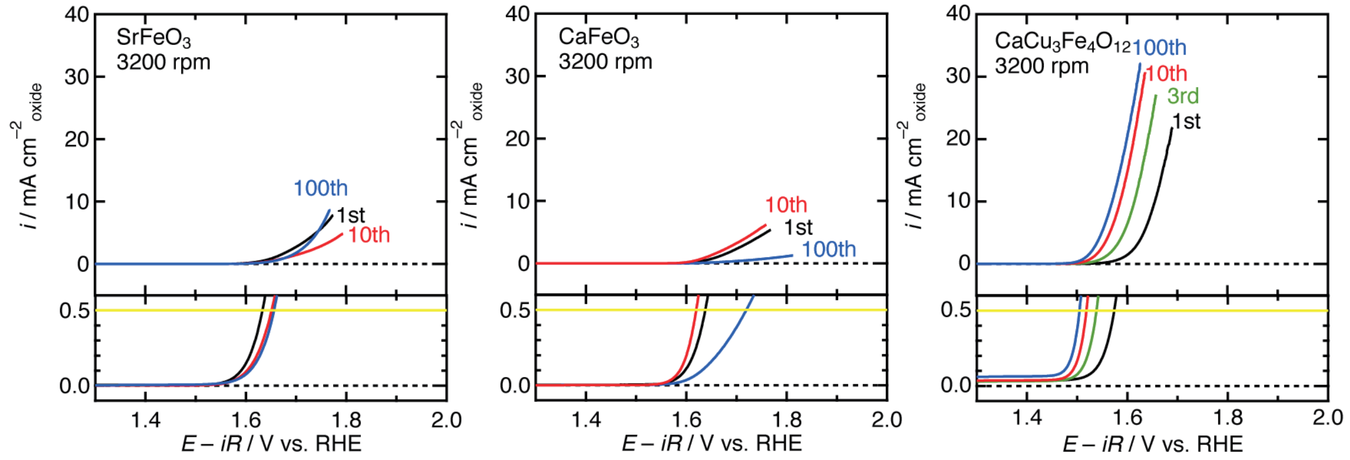

Supplementary Figure 6: **Linear sweep voltammograms of perovskite oxides for 100 cycles.**

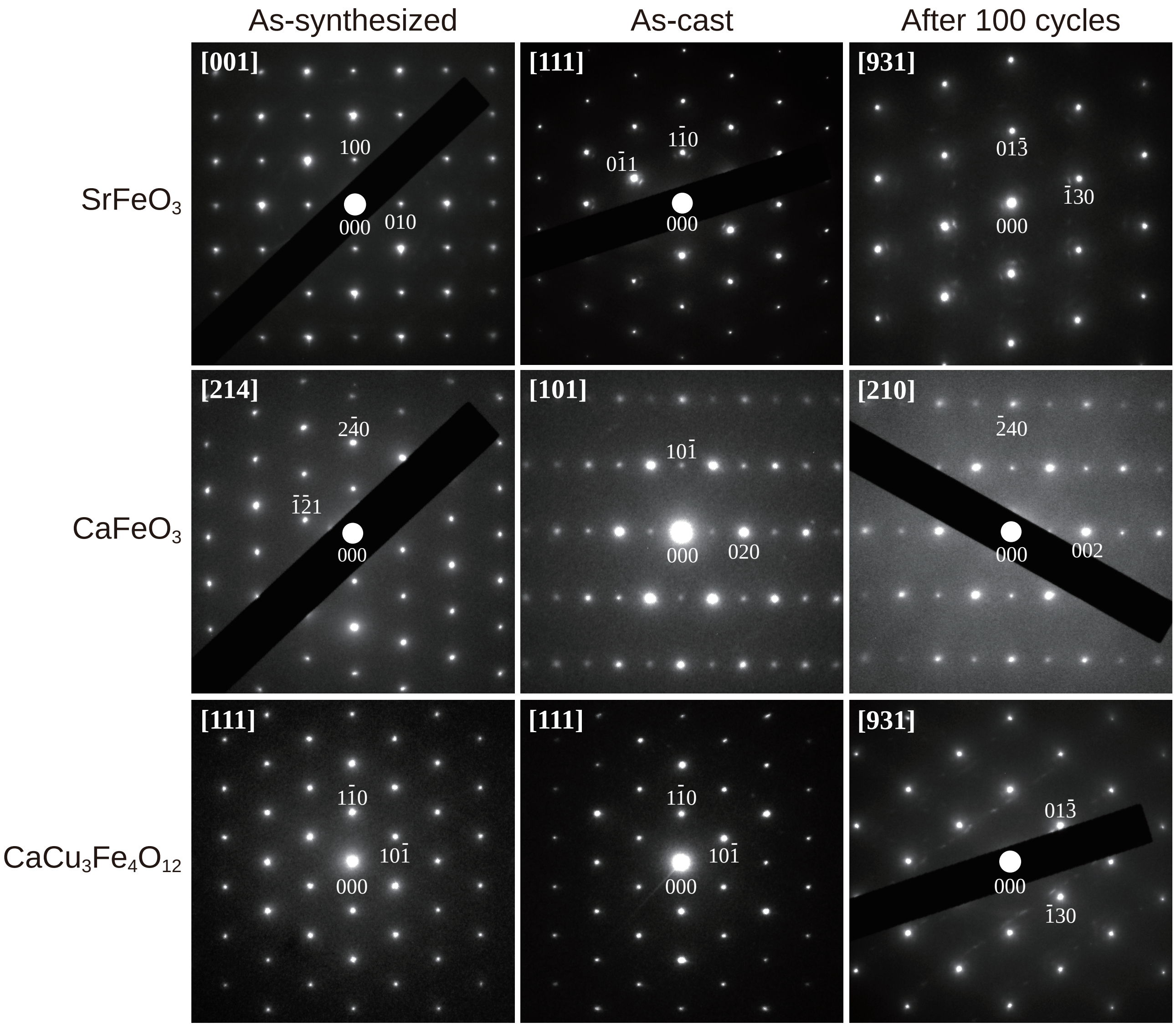

Supplementary Figure 7: **Electron diffraction patterns for provskite oxides before and after 100-cycle OER measurements.** The bulk crystallinity of all these samples are retained after the cast and 100-cycle OER measurements.

Supplementary Table

Supplementary Table 1: **Surface area of oxide samples.** Density ρ, number-averaged diameter, d_{number}, volume-area averaged diameter, $d_{\mathrm{v/a}}$, specific surface area estimated by the SEM images, A_{s}, and specific surface area determined using the BET method, A_{sBET}. All these parameters, apart from ρ and A_{sBET}, were estimated using the particle size distribution indicated by the SEM images.

	ρ/g cm^{-3}	d_{number}/μm	$d_{\mathrm{v/a}}/\mu$m	A_{s}/m^{2}g^{-1}	A_{sBET}/m^{2}g^{-1}
$CaFeO_3$	4.45	2.62	3.89	0.35	0.78
$SrFeO_3$	5.56	1.86	5.40	0.20	0.48
$CaCu_3Fe_4O_{12}$	5.53	1.49	3.41	0.32	0.45
$LaMnO_3$	6.93	0.43	0.54	1.59	2.69
$BSCF_{950}$	5.75	0.72	0.96	1.08	0.67
$BSCF_{1100}$	5.75	8.95	14.4	0.073	0.13
$CuFe_2O_4$	5.39	2.41	4.43	0.25	0.31
RuO_2	7.03	0.21	0.26	3.24	4.88

Supplementary Note 1

Synchrotron X-ray powder diffraction and electron density analysis

The section presents the Rietveld refinement results for $CaFeO_3$, $SrFeO_3$, and $CaCu_3Fe_4O_{12}$ at room temperature, corresponding to **Supplementary Fig. 1**. The synchrotron X-ray diffraction data were collected at the SPring-8 BL02B2 beamline, Japan using a wavelength of λ = 0.41990 Å. Electron density analysis based on the maximum entropy method (MEM) was conducted for $SrFeO_3$ and $CaCu_3Fe_4O_{12}$ (see **Fig. 1b** of the manuscript). The structure parameters obtained from the Rietveld refinement are as follows:

1. CaFeO$_3$: Space group: *Pnma* (No. 62); Lattice constants: a = 5.35184(11) Å, b = 7.53777(16) Å, c = 5.32497(11) Å; Atomic positions: Ca 4c (0.03448(13), 1/4, 0.9935(4)), Fe 4b (1/2, 0, 0), O1 8d (0.2864(4), 0.0331(4), 0.7143(4)), O2 4c (0.4938(5), 1/4, 0.0662(7)); Reliability factors: R_{wp} = 4.731%, R_B = 1.328%, S = 3.0362.

2. SrFeO$_3$: Space group: $Pm\overline{3}m$ (No. 221); Lattice constants: a = 3.85147(5) Å; Atomic positions: Sr 1a (0, 0, 0), Fe 1b (1/2, 1/2, 1/2), O 3c (0, 1/2, 1/2); Reliability factors: R_{wp} = 1.610%, R_B = 0.725%, S = 1.6563. MEM-Rietveld refinement was performed based on 100×100×100 pixels as structure factors.

3. CaCu$_3$Fe$_4$O$_{12}$: Space group: $Im\overline{3}$ (No. 204): Lattice constants: a = 7.29042(8) Å; Atomic positions: Ca 2a (0,0,0), Cu 6b (0, 1/2, 1/2), Fe 8c (1/4, 1/4, 1/4), O 24g (0, 0.1755(3), 0.3072(3)); Reliability factors: R_{wp} = 5.804%, R_B = 2.563%, S = 3.1185. MEM-Rietveld refinement was performed based on 128×128×128 pixels as structure factors.

Supplementary Note 2

Estimation of surface area

In this study, the specific surface area (SA) values estimated from the Brunauer-Emmett-Teller method (BELSORP-max, BEL Japan, Inc.) were used for the calculation of the current density per oxide surface area (mA cm^{-2}_{oxide}), so as to exclude geometrical effects. The measurements were conducted using N_2 gas for $CaCu_3Fe_4O_{12}$, $CuFe_2O_4$, and RuO_2, and Kr gas for $CaFeO_3$, $SrFeO_3$, $LaMnO_3$, $BSCF_{950}$, and $BSCF_{1100}$. In addition, the surface morphologies of the perovskite oxides were observed using scanning electron microscopy (SEM: JEOL Ltd., JSM-6010LA). **Supplementary Fig. 3** shows the SEM images of the synthesized $CaFeO_3$, $SrFeO_3$, $CaCu_3Fe_4O_{12}$, $LaMnO_3$, $BSCF_{950}$, $BSCF_{1100}$, $CuFe_2O_4$, and RuO_2 (Sigma-Aldrich). For reference, the mass-specific SAs, A_s, of the oxide catalysts were estimated based on the diameters of the individual oxide particles, d, which were determined from the SEM images shown in **Supplementary Fig. 3** using a spherical geometry approximation[2]. Here,

$$A_s = \frac{\text{surface area}}{\text{mass}} \approx \frac{\sum 4\pi\left(\frac{d}{2}\right)^2}{\sum \rho\frac{4}{3}\pi\left(\frac{d}{2}\right)^3} = \frac{\sum \pi d^2}{\sum \frac{1}{6}\rho\pi d^3} = \frac{6}{\rho}\frac{\sum d^2}{\sum d^3} = \frac{6}{d_{v/a}\rho} \tag{1}$$

where ρ is the bulk density of the oxides and $d_{\mathrm{v/a}}$ is the volume/area averaged diameter, *i.e.*, $\frac{\sum d^3}{\sum d^2}$. The surface areas of the catalyst oxides are summarized in **Supplementary Table** 1. It was confirmed that the SAs estimated from the SEM images are in good agreement with those obtained from the BET method, for all samples.

Supplementary Note 3

linear sweep voltammograms of perovskite oxides for 100 cycles

Supplementary Fig. 6 shows the linear sweep voltammograms of $SrFeO_3$, $CaFeO_3$, $CaCu_3Fe_4O_{12}$ for 100 cycles, which were obtained by taking the average between the anodic and cathodic scans of the cyclic voltammograms shown in **Figs. 2(d–f)** in the body text. The overpotential (η) of each catalyst was determined from the onset potential, $E_{\rm onset}$ (V vs. RHE); $E_{\rm onset}$ is the potential at 0.5 mA $\rm cm^{-2}_{oxide}$ and $\eta = E_{\rm onset} - 1.23$ (V). The η monotonically increased from 0.40 V (1st) to 0.42 V (10th) to 0.43 V (100th) for $SrFeO_3$, whereas the η slightly decreased from 0.41 V (1st) to 0.39 V (10th), followed by a substantial increase up to 0.49 V (100th) for $CaFeO_3$. This indicates the degradation of $SrFeO_3$ and $CaFeO_3$ catalysts under OER conditions. In contrast, $CaCu_3Fe_4O_{12}$ exhibited a monotonic decrease in η from 0.34 V (1st) to 0.31 V (3rd) to 0.29 V (10th) to 0.27 V (100th), implying the absence of degradation under OER conditions.

Supplementary Methods

Sample preparation

$AFeO_3$ (A = Ca, Sr). $CaFeO_{2.5}$ and $SrFeO_{3-\delta}$ precursors were prepared from stoichiometric mixtures of $CaCO_3/SrCO_3$ (99.99%, RARE METALLIC, Co., Ltd.) and Fe_2O_3 (99.9%, RARE METALLIC, Co., Ltd.) through calcination at 1000 °C for 15 h in air. $CaFeO_3$ and $SrFeO_3$ were synthesized from $CaFeO_{2.5}$ and $SrFeO_{3-\delta}$ with a $KClO_4$ oxidizing agent using a high-pressure and high-temperature (9 GPa and 1200 °C respectively) treatment for 30 min. The obtained powders were washed with distilled water, ethanol, and acetone in that order.

$CaCu_3Fe_4O_{12}$. A precursor was prepared using a polymerization method[3,4]. A mixture of $CaCO_3$ (99.99%, RARE METALLIC, Co., Ltd.), $Cu(NO_3)_2{\cdot}3H_2O$ (99.9%, Wako Pure Chemical Industries, Ltd.), and $Fe(NO_3)_3{\cdot}9H_2O$ (99.9%, Wako Pure Chemical Industries, Ltd.) at a molar ratio of 1:3:4 was dissolved in nitric acid with a five-fold excess of citric acid and one-fold 1,2-ethanediol with stirring. The solution was heated to 300 °C while being stirred, and maintained at this temperature for 1 h to dry. A precursor with a nominal composition of $CaCu_3Fe_4O_{12-\delta}$ ($\delta \simeq 2$) was obtained by firing the dried powder using a furnace at 400 °C for 1 h and 675 °C for 12 h in air with occasional grinding. $CaCu_3Fe_4O_{12}$ was synthesized from a mixture comprising the obtained precursor and a $KClO_4$ oxidizing agent at a molar ratio of 1:1. The mixture was treated at 15 GPa and 1000 °C for 30 min. The obtained powders were washed with distilled water, ethanol, and acetone, in that order.

$Ba_{0.5}Sr_{0.5}Co_{0.8}Fe_{0.2}O_{3-\delta}$. $Ba_{0.5}Sr_{0.5}Co_{0.8}Fe_{0.2}O_{3-\delta}$ was prepared using a polymerization method[3]. A stoichiometric mixture of $Ba(NO_3)_2$ (99.9%, Wako Pure Chemical Industries, Ltd.), $SrCO_3$ (99.99%, RARE METALLIC, Co., Ltd.), $Co(NO_3)_2{\cdot}6H_2O$ (99.9%, Wako Pure Chemical Industries, Ltd.), and $Fe(NO_3)_3{\cdot}9H_2O$ (99.9%, Wako Pure Chemical Industries, Ltd.) at a molar ratio of 5:5:8:2 was dissolved in nitric acid with a five-fold excess of citric acid and one-fold 1,2-ethanediol with stirring. The solution was heated to 300 °C while being stirred, and maintained at this temperature for 1 h to dry. The dried powder was fired with a furnace at 400 °C for 1.5 h and 950 or 1100 °C for 24 h in air to obtain $BSCF_{950}$ and $BSCF_{1100}$, respectively.

$LaMnO_3$. $LaMnO_3$ was prepared using a polymerization method[3]. A stoichiometric mixture of $La(NO_3)_3{\cdot}6H_2O$ (99.9%, Wako Pure Chemical Industries, Ltd.) and $MnCO_3$ (99.9%, Wako Pure Chemical Industries, Ltd.) at a molar ratio of 1:1 was dissolved in nitric acid with a five-fold excess of citric acid and one-fold 1,2-ethanediol with stirring. The solution was heated to 300 °C while being stirred, and maintained at this temperature for 1 h to dry. The dried powder was fired using a furnace at 400 °C for 1.5 h and 1000 °C for 10 h in air.

$CuFe_2O_4$. Cubic $CuFe_2O_4$ spinel oxide was synthesized using the inverse coprecipitation method. An aqueous metallic nitrate salt solution (100 ml, 0.080 M Cu(II), 0.160 M Fe(III)) was prepared by dissolving $Cu(NO_3)_2{\cdot}3H_2O$ (GR grade, Nacalai Tesque, Inc.) and $Fe(NO_3)_3{\cdot}9H_2O$ (GR grade, Nacalai Tesque, Inc.) in deionized water. A sodium carbonate solution (200 ml, 0.350 M Na_2CO_3) for pH control and precipitation was also prepared. These solutions were heated to 70 °C under vigorous stirring. The nitrate salt solution was added dropwise into the sodium carbonate precipitation solution; this procedure is called "inverse coprecipitation", because a precipitation solution

is added into a metallic salt solution in normal coprecipitation. 10 min after all the nitrate salt solution had been added, the resulting suspension was filtered. The filtered precipitate was rinsed with deionized water (*ca.* 300 ml) at 70 °C, and dried in air for 24 h at 80 °C. The obtained carbonate hydroxide precursor was calcined in air at 1100 °C for 12 h.

Supplementary References

---

[1] Blasco, J., Aznar B., García, J., Subías, G., Herrero-Martín & Stankiewicz, J. Charge disproportionation in $La_{1-x}Sr_xFeO_3$ probed by diffraction and spectroscopic experiments. *Phys. Rev. B* **77**, 054107 (2008).

[2] Suntivich, J., May, K. J., Gasteiger, H. A., Goodenough, J. B. & Shao-Horn, Y. A perovskite oxide optimized for oxygen evolution catalysis from molecular orbital principles. *Science* **334**, 1383–1385 (2011).

[3] Kakihana, M. "Sol-gel" preparation of high temperature superconducting oxides *J. Sol-Gel Sci. Technol.* **6**, 7–55 (1996).

[4] Yamada, I., Tsuchida, K., Ohgushi, K., Hayashi, N., Kim, J., Tsuji, N., Takahashi, R., Matsushita, M., Nishiyama, N., Inoue, T., Irifune, T., Kato, K., Takata, M. & Takano, M. Giant negative thermal expansion in the iron perovskite $SrCu_3Fe_4O_{12}$ *Angew. Chem. Int. Ed.* **50**, 6579–6582 (2011).
